# Inner capillary diameter of hypothalamic paraventricular nucleus of female rat increases during lactation

**DOI:** 10.1186/1471-2202-14-7

**Published:** 2013-01-10

**Authors:** Albertina Cortés-Sol, Miguel Lara-Garcia, Mayvi Alvarado, Robyn Hudson, Pere Berbel, Pablo Pacheco

**Affiliations:** 1 Instituto de Neuroetología, Universidad Veracruzana, Dr. Luis Castelazo s/n Col. Industrial Las Animas, Xalapa, Veracruz, 91190, México; 2 Instituto de Investigaciones Biomédicas, Universidad Nacional Autónoma de México, Ciudad Universitaria, México D.F, 04510, México; 3 Departamento de Histología y Anatomía, Facultad de Medicina, Universidad Miguel Hernández, Crta. Nacional, Km-332 s/n, Sant Joan, Alicante, 03550, España

**Keywords:** Inner capillary diameter, Endothelial cell, Neurovascular compartment, Hypothalamus, Lactation

## Abstract

**Background:**

The role of the endothelial cell (EC) in blood flow regulation within the central nervous system has been little studied. Here, we explored EC participation in morphological changes of the anterior hypothalamic paraventricular nucleus (PVN) microvasculature of female rats at two reproductive stages with different metabolic demand (virginity and lactation). We measured the inner capillary diameter (ICD) of 800 capillaries from either the magnocellular or parvocellular regions. The space occupied by neural (somas, dendrites and axons) and glial, but excluding vascular elements of the neurovascular compartment was also measured in 100-μm^2^ sample fields of both PVN subdivisions.

**Results:**

The PVN of both groups of animals showed ICDs that ranged from 3 to 10 microns. The virgin group presented mostly capillaries with small ICD, whereas the lactating females exhibited a significant increment in the percentage of capillaries with larger ICD. The space occupied by the neural and glial elements of the neurovascular compartment did not show changes with lactation.

**Conclusions:**

Our findings suggest that during lactation the microvasculature of the PVN of female rats undergoes dynamic, transitory changes in blood flow as represented by an increment in the ICD through a self-cytoplasmic volume modification reflected by EC changes. A model of this process is proposed.

## Background

The blood brain barrier (BBB) in the central nervous system (CNS) regulates the passage of nutrients, essential components and metabolic constituents between the blood stream and the parenchymal tissue. Although local neural activity is known to promote local blood supply and is the basis of the so-called blood-oxygen level gradient/dependency (BOLD) [1-3], the regulation of local blood flow in the parenchyma of the CNS is not well understood. Contraction of pre-capillary arterioles has been suggested but so far without clear demonstration [4]. Capillaries of the CNS do not have smooth musculature but contractility of pericytes associated with the capillaries of peripheral tissue such as the retina, cardiac and skeletal muscles has been observed [5-9], suggesting that contraction of these cells in the CNS can be a mechanism of blood supply regulation [10-12]. However, the finding that pericytes of the CNS lack the α-actin protein isoform found in contractile cells [13], appears to exclude their participation in CNS capillary contraction. Angiogenesis described during brain development [14] has also been suggested as a mechanism of blood flow increment, although without clear support in normal adulthood or non-pathological contexts [15-18]. The endothelial cells (EC), pericytes and basal lamina (anatomical constituents of the BBB), together with elements of the neurovascular compartment such as neurons, astrocytes, and other glial cells, adapt themselves to maintain homeostasis that promotes tissue survival [19,20]. Although the endothelial cell can be considered as an element which plays an important role in blood flow regulation its participation has been little analyzed, at least partly due to the intrinsic difficulty of its individual staining in capillaries of the CNS [21-23].

However, in order to better understand the mechanisms that occur during local blood flow changes in the CNS parenchyma, the role of capillary morphology needs to be considered as an important issue. Electron microscopy (EM) has provided knowledge of the capillary wall constituents [24,25], but the small spatial range of this method is a critical and important limitation of this powerful tool, e.g. the size of a capillary endothelial cell is still not known. Despite attempts to obtain markers enabling CNS capillary imaging or visualization of capillary networks through extracellular tracers, fluorescent markers, diamine benzidine reaction, immunocytochemistry as well as brain arterial injection with black ink or venous plastic infusion [1,15,22-24,26], a complete view of the morphology of CNS capillaries still is lacking. In this respect, recently we proposed an alternative histological tool focused on detecting the relative space occupied by the capillary endothelial cell [27,28]. This visualization through light microscopy is based on two facts: first, the optical effect that reflecting light produces in fixed and unstained capillaries, and second, evidence provided by EM [24,25] showing that the internal wall of CNS capillaries is exclusively constituted by the endothelial cell. Using this procedure we found that changes in capillary luminal area can be detected indirectly via changes in the inner capillary diameter (ICD).

With the aim of investigating the participation of the capillary endothelial cell in the regulation of blood flow fluctuations that occur in response to changes in neuronal activity, the present study was directed to quantifying the internal diameter of capillaries in the anterior hypothalamic paraventricular nucleus (PVN) in female rats. We chose this nucleus since several of its physiological aspects but not its microvasculature have been intensely studied [29-35]. For example, it is well known that the magno- and parvocellular regions via a neuroendocrine reflex are involved in oxytocin (OT) production and release in response to mammary gland sensory stimulation [36,37], and that the effect varies according to the reproductive phase of the female rat [33-35,37]. Thus, we decided to measure and compare the capillary internal diameter during the diestrous phase of virgin rats and during the lactation period of mother rats. To ensure that the sensory information received by the two groups was significantly different, mammary glands of the mother rats were stimulated by their pups suckling for two weeks of lactation, while virgin rats had no stimulation.

## Results

### General microvasculature characteristics

**Figure 1:**
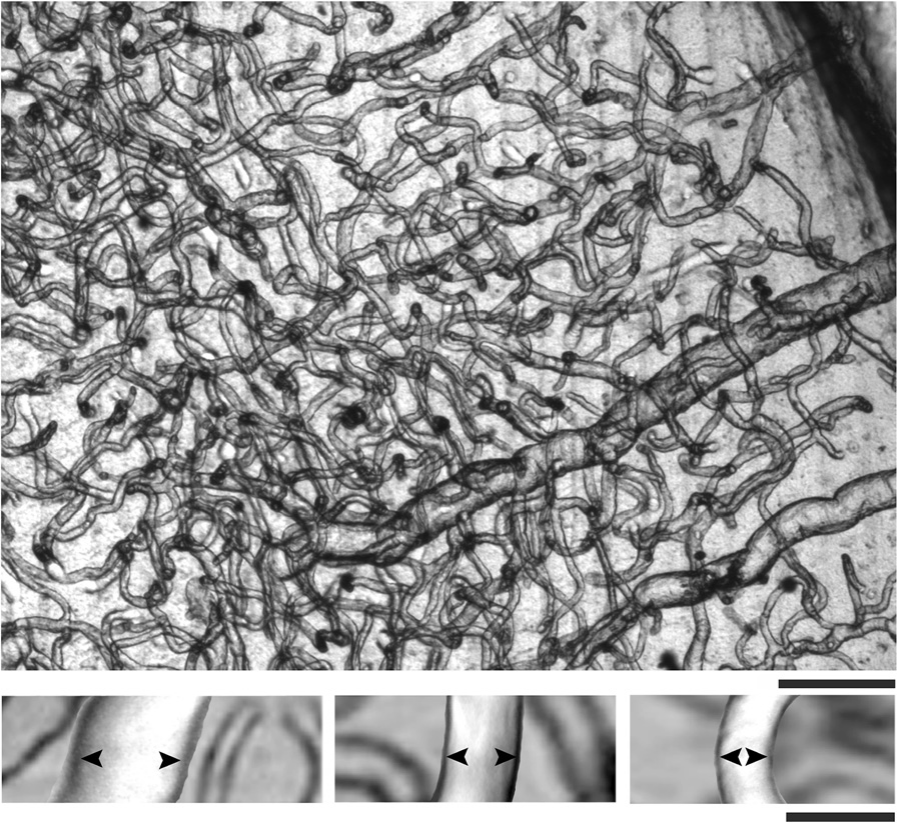
**Blood vessels of the rat brain.** Upper photomicrograph shows a representative unstained brain section illustrating networks of highly interconnected blood vessels. Lower photomicrographs show the inner diameter size (arrowheads) of three focused capillaries. Scale bars: 100 μm and 10 μm respectively.

**Figure 2:**
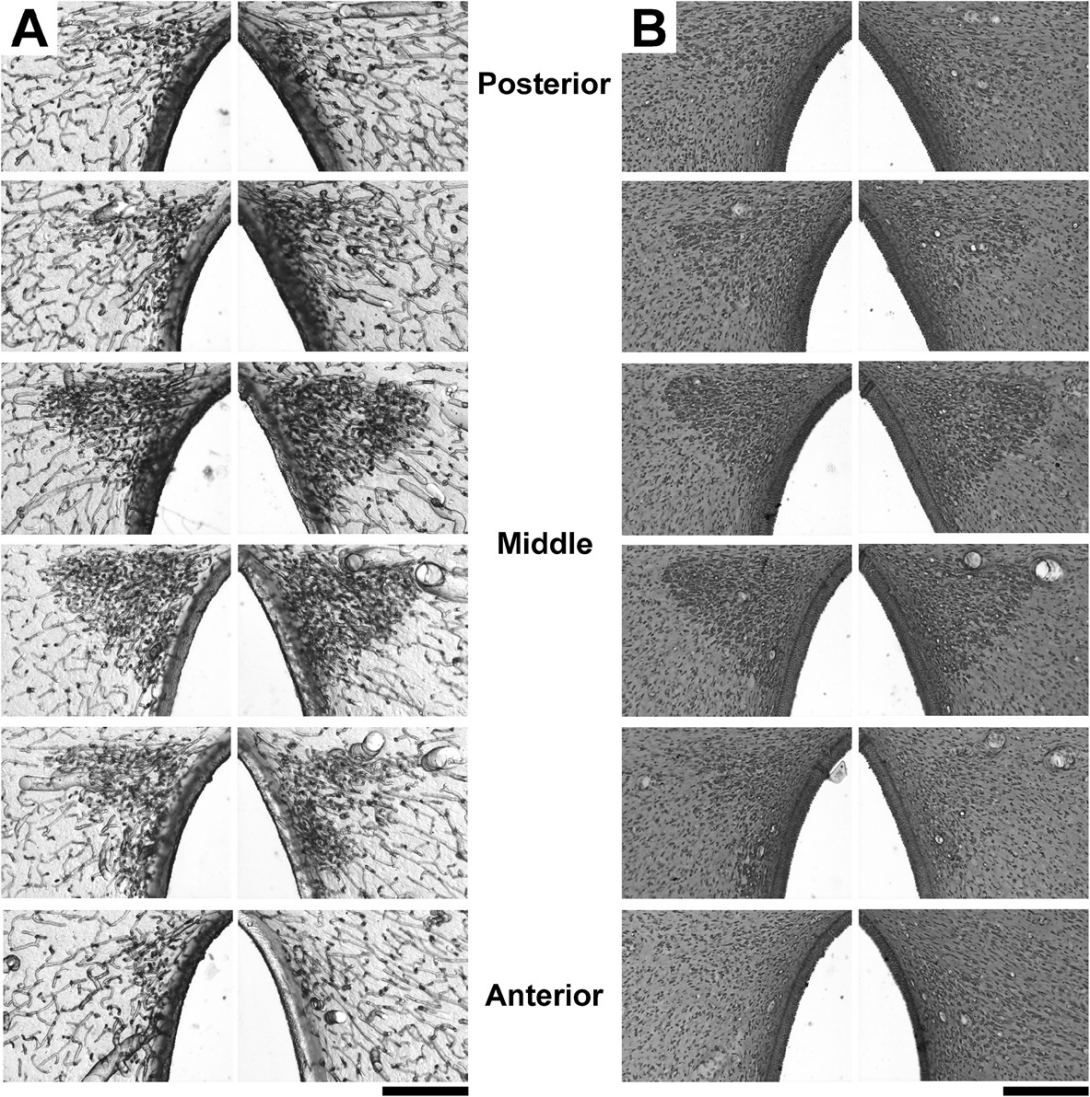
**Photomicrographs of transversal sections through the anterior hypothalamus of a female rat. A**) High capillary density in the hypothalamic PVN observed in six serial 100-μm unstained sections. **B**) The same six sections after cresyl violet staining; note that capillaries are not visualized but stained neurons can be seen (for details see text). Scale bar: 200 μm.

**Figure 3:**
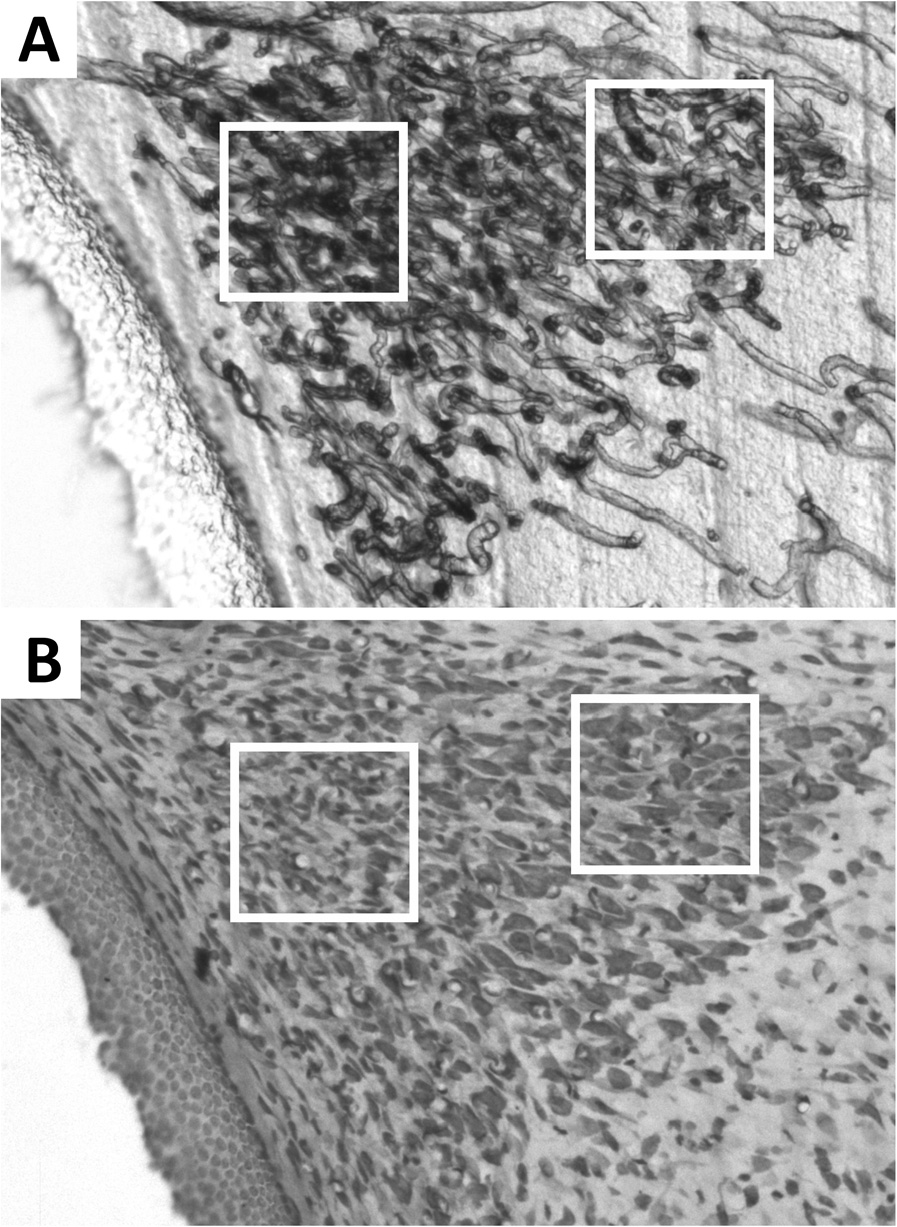
**Photomicrograph of a middle transversal section of the PVN from a female rat. A)** High capillary density visualized in the still unstained section; note that the border of the nucleus is easily identifiable. **B**) The same section after cresyl violet staining; capillaries no longer visible but stained neurons can be seen. The 100 x100 μm squares delimit sampling fields within the parvocellular (left) and magnocellular (right) subdivisions.

Unstained brain sections from all animals were characterized by networks of highly interconnected blood vessels with a wide range of calibers (Figure 1). In particular, sections from the anterior hypothalamic region showed an area of high capillary density corresponding to the PVN [38]; clearly seen in six serial sections with an anterior-posterior length of approximately 600 μm (Figure 2A). This conspicuous density of capillaries could no longer be visualized after cresyl violet staining (Figure 2B), since application of the cover slip and permount modified the optical effect that reflecting light produces on the capillaries, thus impeding their visualization. However, the stained neurons enabled us to confirm the exact PVN location as well as the location of the magno- and parvocellular regions (Figure 3). 

### Inner capillary diameter (ICD)

**Figure 4:**
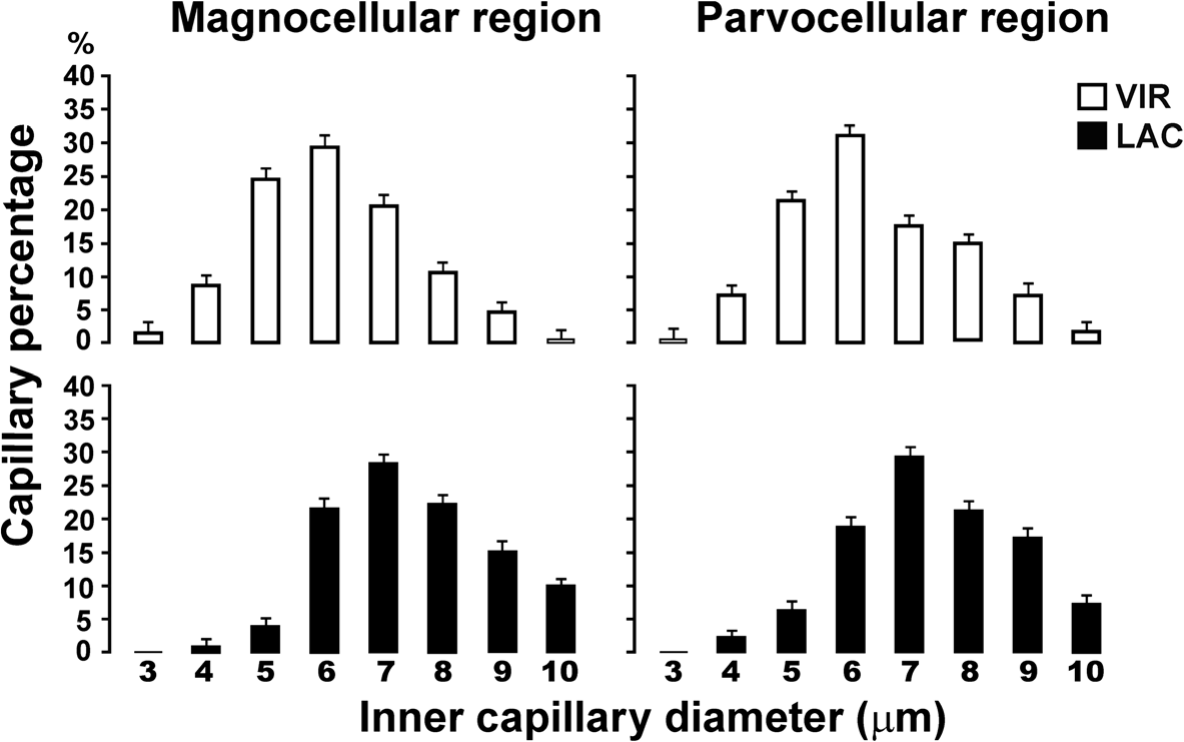
**Percentage distribution of inner capillary diameters in the PVN of virgin and lactating rats.** Upper panels (white bars) represent data obtained from the magno- and parvocellular regions of the virgin group. Lower panels (black bars) represent data from the lactating group, which showed a tendency to have capillaries with larger internal diameters (for more details see text). Means ± SEM

When for the virgin animals in diestrous phase (VIR group) the ICD (μm) values from the magnocellular subdivision of the left PVN were compared with those of the right PVN there was no difference [F(7,64)=1.71, P=0.12]. Similarly there was no difference when the values from the parvocellular subdivision of the left PVN were compared with those of the right PVN [F(7,64)=0.88, P=0.52]. Also in the mother lactating animals (LAC group) the ICD values of the left and right magnocellular subdivisions did not differ [F(7,64)=0.14, P=0.95], nor in the left and right parvocellular subdivisions [F(7,64)=1.5, P=0.18]. Therefore, the data from each cellular subdivision were combined. As shown in Figure 4, the VIR group presented ICDs ranging from 3 to 10 μm in both regions, with most capillaries located in the 6-μm range, followed by the 5- and 7-μm ranges, respectively. When the percentage values of the ICD magnocellular and parvocellular subdivisions were compared, they did not differ significantly [F(7,64)=0.69, P=0.67]. In contrast, the LAC group showed an ICD distribution that ranged from 4 to 10 μm in both regions, with most capillaries located in the 7-μm range followed by the 8- and 6-μm ranges, respectively. Again, when the percentage values from the two regions were compared, no difference was found [F(7,64)=0.34, P=0.93]. Thus, as seen in Figure 4, the LAC group showed a tendency towards a greater percentage of capillaries with large ICD.

**Figure 5:**
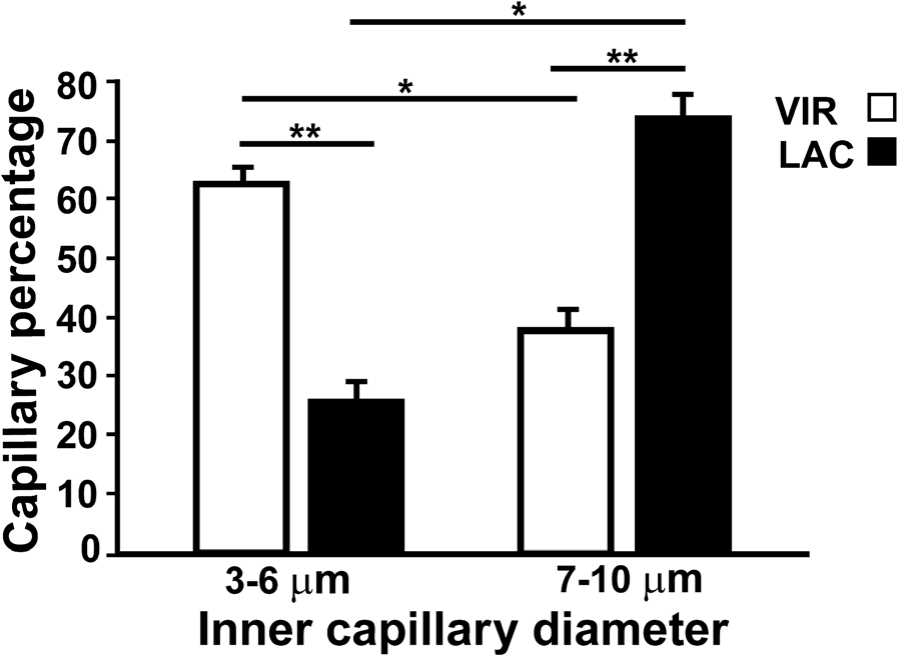
**Percentage of capillaries grouped in bins of 3-6 μm and 7-10 μm internal diameter from virgin (VIR) and lactating (LAC) animals.** The VIR group as compared to LAC group showed a significantly greater percentage of capillaries in the 3-6 μm bin, while in the 7-10 μm bin the significantly greater percentage was for the LAC group. Means ± SEM. F (7, 32)= 23.05; *P < 0.05.

To clarify this observation and since there were no significant differences between the measurements obtained in the two PVN subdivisions of either group, we decided to compare the data between the two experimental conditions by grouping the ICD measurements into two capillary ranges: with small (3-6 μm) and large (7-10 μm) diameters (Figure 5). This confirmed that the LAC group had a significantly higher percentage of large diameter capillaries compared to the VIR group [One-way ANOVA: F(7,32)=23.05, *P < 0.05].

### PVN neurovascular compartment

**Figure 6:**
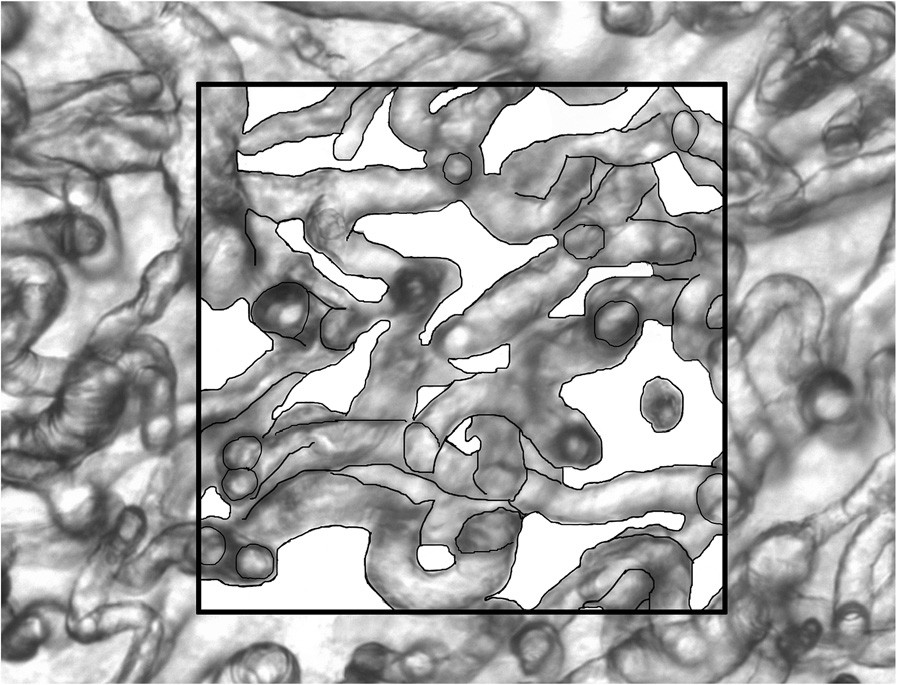
**Example of a 100x100-μm delimited field from the magnocellular region of the PVN.** The white painted area represents the space occupied by neuronal and glial elements of the PVN neurovascular compartment.

When values (μm^2^) for the space occupied by neural (soma, dendrites and axons) and glial (white area Figure 6), but excluding vascular elements of the neurovascular compartment were compared between PVN magno- and parvocellular subdivisions from the VIR and LAC groups, we found no significant differences between the two groups [F(3,16)=2.69, P=0.08]; neural and glial elements (white area) occupied approximately 30% of the space in all the sample fields analyzed.

## Discussion

Whereas the physiology and neuronal characteristics of the PVN have been widely investigated [29-35], its microvasculature has been much less so [29,39,40]. Morphological changes such as glial retraction, neuronal remodeling, c-Fos expression and electrophysiological activity increment during lactation have been reported [35,41-43], and there is no doubt that greater synthesis and release of OT from both the PVN and supraoptic nucleus occurs [33-35]. This could reflect an increment in the local neural activity of these structures that in turn promotes an increment in local blood flow [1-4]. In the present study our results suggest that, at least in the PVN, local blood flow increment could be via an increment in the ICD rather by an increase in outer capillary diameter or angiogenesis. Our findings showing that the area occupied by the neural and glial elements of the neurovascular compartment did not change in virgin compared to lactating rats, suggest that angiogenesis is not present. In addition, it has been suggested that components of the capillary basal lamina such as laminin, cablin, fibronectin and type IV collagen among others [44-47] prevent the outer EC diameter from expanding.

**Figure 7:**
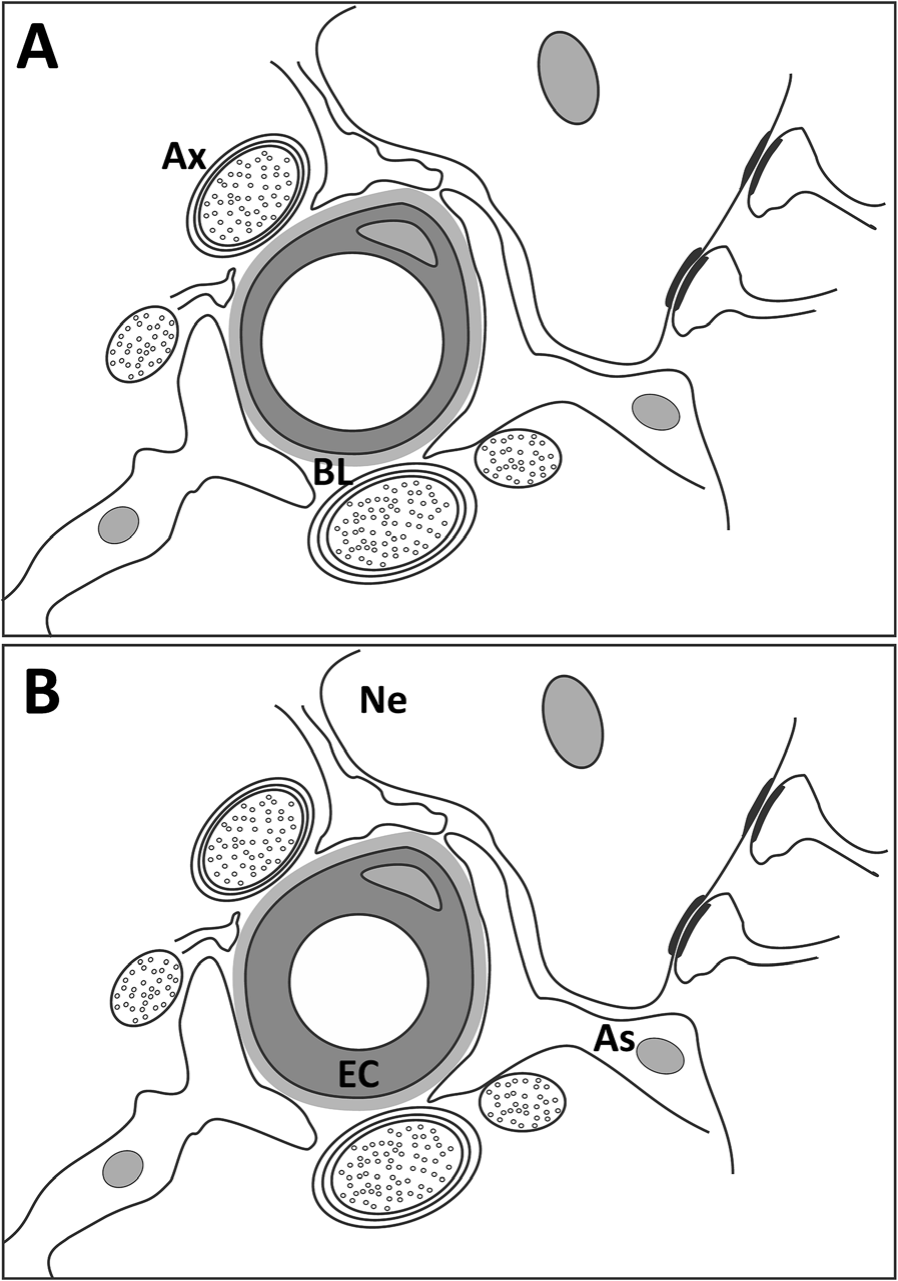
**Diagram illustrating our model of the organization of a capillary in the CNS. A**) Size of the capillary luminal area when the endothelial cell cytoplasmic volume is reduced. **B**) The capillary luminal area when the endothelial cell cytoplasmic volume is increased. Note that the outer capillary diameter does not change (for more detail see text). As, astrocyte; Ax, axon; BL, basal lamina; EC, endothelial cell; Ne, neuron.

In relation to our proposal that ICD increment promotes increases in blood flow, we mention the following: Local blood flow regulation in the parenchyma of the CNS is not well understood [4-7,10-12]. However, capillary contraction caused by swelling of EC with no change in the external diameter of the capillary has been previously suggested as an active mechanism of local capillary constriction in peripheral tissue [48]. Thus, in accordance with our findings of significant changes in the ICD of PVN microvasculature in the LAC group, we take up this idea again to offer an account of how fast, regional, and reversible control of blood supply in the CNS might occur. Changes in the ICD might take place via modifications of EC cytoplasmic volume; when this decreases the capillary lumen increases (Figure 7A), and the time needed for cytoplasmic transport of substances from the luminal to the abluminal side of the membrane decreases. It is known that the reduction or increase in either local blood supply or the bidirectional “blood ↔ parenchyma” transport of metabolic constituents depends upon the EC which, together with the basal lamina, constitute the capillary wall [25,47]. Accordingly, when the cytoplasmic volume increases, the capillary lumen decreases (Figure 7B), causing cytoplasmic transport of metabolites from the luminal to the abluminal side of the membrane to be slowed. There are a wide variety of factors that modify intracellular osmolarity, thereby promoting changes in cytoplasmic EC volume. Organic osmolytes, taurine, cyclosporine, water, some hormones such as vasopressin (VP) or OT, and free radicals have all been implicated in mechanisms of cell volume regulation [49-53]. Thus, it is possible that neurons and/or astrocytes could modulate their own capillary blood supply as well as the transport and supply of metabolically important substances via such factors. 

In addition, it is currently thought that transport of water-soluble molecules across EC is accomplished by the continuous formation of plasmalemma vesicles followed by detachment and fusion to the membrane on the other side of the cell or by transport of cytoplasmic vesicles moving from one surface to the other without membrane fusion [47,54,55]. Either of these mechanisms of intracellular transport could be present in the capillary EC and depending on the luminal-abluminal distance the cytoplasmic transport time could be faster or slower. This could represent a dynamic and plastic mechanism by which constantly changing neuronal needs are rapidly met. As physiological changes associated with chronic dehydration, hemorrhage, stress condition as well as pregnancy and parturition [33,56], are associated with the synthesis and release of OT or VP from the PVN and supraoptic nucleus, then, based on our results, we should not reject the idea that a similar dynamic mechanism of blood flow regulation in response to such stimuli might occur.

Finally, it is well-known that during lactation progesterone (P4) and prolactin (PRL) reach high levels in the blood stream [33]. Since PRL has been implicated in OT release [57,58], this hormone could be a potential candidate to participate in PVN blood flow regulation.

## Conclusions

Our findings suggest that during lactation the microvasculature of the PVN of female rats undergoes dynamic, transitory changes in blood supply represented by an increment in the ICD through self-cytoplasmic volume modification reflected by EC changes. This is relevant to consider from the pharmacological point of view, since changes in cytoplasmic volume in the EC could help to increase blood flow and in this way facilitate the metabolic exchange between capillaries and the CNS parenchyma.

## Methods

### Animals, groups, and experimental rationale

All experimental procedures comply with the requirements of the Institutional Ethical Committee of the Universidad Veracruzana, which is in agreement with the official Mexican regulations (NOM-062-ZOO-1999). In addition the international guidelines for the production, care, and use of laboratory animals from The Society for Neuroscience, USA, were considered.

We used adult female Wistar rats, bred and maintained in the vivarium at the Instituto de Neuroetología, Xalapa, Veracruz. They were kept on a 14:10 light/dark cycle with free access to rat chow (Harlan, Mexico) and water. Given that our aim was to investigate possible changes in the microvasculature of the PVN under different demands for OT secretion, we divided the animals randomly into two groups: virgin females and mother rats. The virgin females were housed in two collective cages (44 × 34 × 20 cm) with 5 animals in each. Their estrous cycle was determinated by vaginal smears and after two regular consecutive estrous cycles, 5 rats were chosen to be perfused during their diestrous-2 phase (n=5) (VIR group) (they were approximately 140 days old). Mother lactating rats (n=5) (LAC group) were kept with eight pups each in individual cages (37 × 27 × 17 cm). On the 14^th^ day of lactation pups were separated from their mother and maintained in an incubator during 4 h, returned to their mother for a 30-min suckling period, after which the mother was perfused (also when approximately 140 days old). The pups were placed with a nursing dam to minimize animal sacrifice.

### Histology

Under an i.p. overdose of sodium pentobarbital anesthesia (40 mg/kg), animals were transcardiacally perfused using 50 ml of a 0.9% saline solution and 300 ml of fixative at 4°C (4% paraformaldehyde, 1% glutaraldehyde, 0.002% calcium chloride and 3.2% sucrose, diluted in 0.1 M phosphate buffer pH 7.4). To minimize the effects of anoxic conditions and perfusion pressure on vessel diameters, the perfusion procedure was standardized in all animals (Masterflex pump 77200, 100 strokes-min, 0.25 ml/stroke calibration). Brains were rapidly removed into fresh fixative for 12 hr. They were then mounted on a vibratome (EMS OTS-4000) and 100-μm serial transversal sections were cut. Sections at the anterior hypothalamus level [38] were collected in 0.1 M phosphate buffer, mounted on gelatinized slides and dried at room temperature for immediate microscopy analysis (sections remained unstained) (Figures 2A and 3A). Afterwards, sections were stained using the Nissl technique (0.04% cresyl violet, pH 3.4) and prepared with a cover slip and permount for histological confirmation of the PVN borders as well as for identification of the magno- and parvocellular regions (Figure 3B).

### Inner capillary diameter (ICD)

The unstained sections were examined at 20X and 40X magnification under a light microscope (Olympus CX31) equipped with a video camera (CoolSNAP-Pro, MediaCybernetics, Inc) for image storage. The condenser lenses were aligned to assure that Koehler illumination was optimal. Measurements were made using Image-Pro plus v.6 software (MediaCybernetics, Inc). The capillaries were clearly visualized, as well as their ICD (Figure 1 lower panel). When the ICD was larger than 10 μm, we considered it as a vessel instead of a capillary [59] and excluded it from our analysis. In the sections corresponding to the PVN middle area (see Figure 3), 20 capillaries from each magno- and each parvocellular subdivision from both sides were randomly selected and focused to measure their ICDs. A total of 80 measurements per animal were obtained and thus 400 measurements for each group.

Considering the criteria used in other studies where a morphometric analysis was done [60,61] the percentage distribution of capillaries was organized according to individual ICDs for each PVN region (magno- or parvocellular) and for each group of rats. Also, in the present work the mean ICD percentage distribution was calculated and categorized according to bins, with an ICD range of 3-6 and 7-10 μm each.

### PVN neurovascular compartment

After the analysis of ICDs, the area occupied by the neural and glial elements of the PVN neurovascular compartment in the magno- and parvocellular regions within each group (10 fields per group) was measured in a field of 10, 000 μm^2^ by painting them out so as to exclude the intravascular space (Figure 6).

### Statistical analysis

The percentage of PVN capillaries distributed according to the ICD size and the space occupied by neural and glial elements of the neurovascular compartment were both analyzed using a one-way ANOVA. Tukey's HSD post-hoc test was applied when necessary and statistical significance set at P < 0.05. Data were analyzed using SPSS Statistics v.18 software (IBM). Descriptive statistics are expressed as means ± SEM.

## Abbreviations

BBB: Blood brain barrier; CNS: Central nervous system; EC: Endothelial cell; EM: Electron microscopy; ICD: Inner capillary diameter; LAC: Lactating group; OT: Oxytocin; PRL: Prolactin; PVN: Hypothalamic paraventricular nucleus; VIR: Virgin group; VP: Vasopressin.

## Competing interest

The authors declare that they have no competing interests.

## Authors’ contributions

ACS participated in the design of the study, performed the experiment, processed and analyzed the data, and drafted the manuscript. MLG provided assistance with data processing and analyses. MA provided recommendations for the experimental design and analyses. RH and PB provided insight, recommendations and edited the manuscript. PP conceived the study, supervised the experimental design and the coordination of all parties involved with the study, and edited the manuscript. All authors read and approved the final draft.
